# Cynanche Laryngea Chronica, or Chronic Laryngitis

**Published:** 1838-01-03

**Authors:** N. Chapman

**Affiliations:** Professor of the theory and practice of Physic, in the University of Pennsylvania


### CYNANCHE LARYNGEA CHRONICA,

### OR CHRONIC LARYNGITIS.

By N. Chapman, M. D., *Professor of the theory and practice of Physic, in the University of Pennsylvania.*

It may be proper to premise, in entering on the consideration of the chronic lesions of the windpipe, that they do not uniformly bear a very exact resemblance to either of the acute affections of this part of which I have just treated.*

Cases are presented, approaching more nearly to the one or the other, and sometimes, we meet with varieties differing from both. The term Laryngitis, therefore, I adopt on this occasion, in conformity with the conventional understanding of it, to express a series of chronic degenerations of the respiratory tube, and not in its limited and strict sense.

By those who are studious of nosological nicety, a division has been made between the lesion of the larynx and the trachea, and each has been erected into a separate disease. That those portions of the windpipe may independently suffer, is sufficiently probable, though the larynx more commonly, and its affection being of greater danger, has the stronger claims to attention. The distinction, however, is not important, having very slender practical bearing, and hence will not be rigidly observed by me.

Of these lesions, the simplest and least formidable, is that familiarly termed hoarseness, where the voice is rough and raucal, or low and feeble, rarely attended with constitutional disturbance; the only serious inconvenience experienced, being an impaired phonation. The duration of such a state may be from a few weeks, or months, to years, or even endure throughout a long life, without any sensible mitigation or aggravation; in proof of which, I have an acquaintance, who for thirty-seven years has not been able to raise his voice above a whisper, and continues to enjoy robust health. Cases however, of this sort do occur, which after an indefinite period, are excited into some activity, and become progressively developed.

Laryngitis proper, usually approaches insidiously, commencing for the most part with huskiness of the fauces, and slight difficulty of swallowing, considered at first, as merely a trivial soreness of throat. But sooner or later, to an increase of these affections, are added a short, dry, worrying cough, hoarseness, pain and embarrassment in speaking; the voice when attempted to be elevated, giving way, followed by shortness of breath, and considerable fatigue, or even temporary exhaustion.

The voice too, independently of such exertions, may be affected. It is usually more hoarse when the stomach is empty, and is sensibly improved by eating. Nor is the deterioration of it less, by an exposure to a temperature materially different from that, in which the individual is habituated; and it is not a little curious, that the effect is sometimes more immediate and greater, by a transition from a cold to a heated apartment, than the reverse.

Now also, uneasiness in the larynx is complained of, rather of stiffness than tenderness, which is speedily converted into a sense of prickling, or stinging, attended by a constant propensity to gulp, and other efforts, as it were, to get rid of something irritating about the fauces or glottis, productive occasionally of paroxisms of spasmodic or convulsive cough, with a peculiar wheeze in making a deep inspiration. On pressure of the larynx, pain is betrayed, though not uniformly, and, indeed, instances have been met with in which several of the preceding symptoms were wanting, or very faintly expressed, and especially those most prominently characteristic of an affection of the windpipe.

The lesion here, for a considerable period, seems to be confined chiefly to the throat, or at least, the complaints are referred to it, and under such circumstances, there is mostly derangement in the digestive functions, denoted particularly, by the sense of gastric oppression, acrid, sour, scalding eructations, constipation, or vitiated alvine discharges, the tongue coated in the centre, and florid at the tip and edges, and with a cough, rather of a nervous or stomachic, than pulmonary character. Examining the throat at this time, different appearances are exhibited, sometimes the vessels are injected and turgid only, and on other occasions, the surface is rough, granulated by the prominency of the cryptœ, or mucous follicles, or perhaps it is delicately inflamed, with an apthous ulceration, or more intensely so, the whole of the soft parts being even tumid, or the tonsils are merely hypertrophied, or there is an elongation of the uvula, either thickened and bloated, or attenuated and relaxed.

No further affection of the system seems to exist at this early period than some irritation of the pulse, and softness and flaccidity of the integuments, with diminution of muscular power, and still oftener of mental energy. The disease, however, henceforward, becomes rapidly evolved, by an exasperation of some of the preceding, and the disclosure of new and less equivocal symptoms, till it reaches its maturity. Embarrassment of breathing now prevails, characterized by a long inspiration, effected by violent muscular exertion, the expiration being comparatively shorter, and less laborious. During the inspiratory efforts, there is a shrill sound from constriction of the glottis,—the voice is very rough and hoarse, or lost in a low indistinct whisper. Dyspnœa of a spasmodic nature periodically returns, and which is sometimes of extreme violence. Cough and incessant weazing, with an impeded expectoration, are nearly constant attendants—the sputa of ropy or thin mucus, sometimes mixed with puriloid or purulent matter, are brought up by hawking, and in these irritating exertions a spasm in the windpipe is excited, which may prove fatal.

The case however, as often lingers along, imitating the career of genuine consumption, of which indeed, it constitutes a variety, having the title of *phthisis laryngitis.* It is under these circumstances, that pain, heavy and obtuse, or more acute, is complained of in the chest, the primary irritation

* Cynanche trachealis and Laryngitis.

involving the lungs, succeeded ultimately by the usual evidence of hectic fever, frequent pulse, hot skin, flushed cheeks, dry and florid tongue, occasionally purulent expectoration, nocturnal sweats, colliquative diarrhœa and extreme emaciation. Towards the close, there is much aggravation of the difficult respiration, and occasionally such vehement fits of coughing as to induce strangulation, sometimes fatal, though more frequently terminating by the disengagement of tenacious mucus, or of pieces of coagulable lymph, or carneous like substances, coloured by blood, or mixed with pus.

The duration of the disease varies from a few weeks to several years.

Chronic laryngitis is chiefly confined to middle and more advanced age, seldom occurring prior to puberty, and is more incident to the male than female sex. By Frank, of Vienna, we are indeed told, that women are entirely exempt from it. But this is contradicted by respectable authorities, with whom I concur, having seen several cases of it in females, though I still suspect it is, comparatively, a rare event.

It has become a very common notion of late in this country, that of all denominations of persons, clergymen are most subject to the disease, and much exegetical speculation indulged in regard to the fact. That it is of very frequent occurrence among them, as are also the other affections of every part of the pulmonary apparatus is unquestionable. Nothing, however, do I know in their habits, or occupations, to dispose them more to such attacks than various other classes of people, and especially the members of the professions of the law, and medicine. The pleadings of the bar, are certainly as often as loud and as long, as the preachings of the pulpit, and the air of a crowded Court house not less impure than that of the most thronged church, or conventicle.

As to us, we are subjected to a tenfold exposure to the most operative of the causes of this and similar diseases—to an equal intensity of study—to the cares and responsibilities of an arduous calling—to interruptions of rest—to the inclemencies of weather by day and by night—dry and wet, hot and cold, the fœtor of hospitals and dissecting rooms, and some of us poor lecturers, I am sure, are not sparing of our lungs or windpipes. The true explanation I suspect of the proneness of clergymen to the disease, was given under the head of Hemoptysis.*

It seems to me, that the predisposition to chronic laryngitis, or, at least, to the most prominent and intractable species of it, is laid in the lymphatic temperament, coupled with a false nutrition—that precise condition which leads to the production of tubercular phthisis, and the strumous degenerations,—though it may originate in any bad habit of body, particularly in irritated and heated states of the stomach, or depravations of any of the chylopoietic viscera.

*It was stated on that occasion, that clergymen are singularly liable to hœmorrhage, and other affections of the lungs, and such indications being early betrayed, they naturally were solemnly impressed with the uncertainty of life, became pious, and devoted themselves to the holy ministry. To defect of constitution and not to their office, was this liability therefore to be ascribed.

Not an instance have I met with of its resulting from an acute attack, which, however, is inconclusive against its happening, the contrary indeed, having been affirmed by writers whose reports are entitled to attention.

Commonly, I think, it may be assigned to very much the same causes, as the the acute form of it operating more slowly, and with less intensity on a feebler, or decayed, or contaminated constitution. Yet it is otherwise induced. Cases of the disease have been traced to neglected or imperfectly cured rubeola, scarlatina, and other exanthemata, or to chronic eruptions repelled, particularly tetter, and still oftener to an extension of phlogosis, or ulceration of the fauces, either common, or scrofulous, syphilitic, mercurial, scorbutic, &c.

Even a more prolific source, according to my observations, is an elongation of the uvula, which though it may be an effect of the disease, most assuredly also, excites it, as repeatedly witnessed by myself.

An irritation from neighboring tumours, or abscesses, or aneurisms, has also sometimes caused it, on the testimony of credible authorities, and we have recently been told, that caries of the teeth has had in the same mode, a similar effect. That it very frequently exists, as an incident of tubercular phthisis, is well ascertained. The very first indication of that disease, may be hoarseness, and other signs of laryngitis, and here, the lungs are secondarily involved. No less true is the reverse, that it proceeds from essentially the same morbid condition of the lungs reflected on the larynx. It may be observed in the progress of phthisis, that the upper part of the windpipe becomes sore, and I have known it many times to be so much affected, as to constitute real laryngitis. This might be presumed, independently of any direct proof of the fact, which however, we have in ample dissections, shewing such a state of the larynx in a large proportion of cases.

By adverting to the established sympathy between the two sections of the pulmonary apparatus, through which an irritation at either extremity is felt by the other, it may be conceived how this reciprocity of mischief is produced.

As occasioning of the disease, a great deal is imputed to an exercise of the vocal organs, by speaking or singing, of which, when regulated with any degree of moderation, and particularly, in the practice of the sacred profession, I must again express my doubts. But the affection really existing, it is very intelligible, how such exertions may exasperate it, and in this view only, I am inclined to believe they prove baneful. The use of any organ is preventive of morbid aggression, by the corroboration imparted, and acquires susceptibility to it in the ratio of its indolence, or inactivity. Becoming disordered however, employment serves to promote the injury, and to weaken the capacity of resistance to its extension, or aggravation. Nevertheless, I do not wish to be understood as denying that inordinate efforts of the kind in vehemence or duration may not conduce to the disease, and more particularly where there is any tendency to it.

It will be well in deciding on the nature of the case, to examine in the first place, very carefully, every part of the throat, to ascertain its condition. As mentioned on a recent occasion* several of its lesions have a considerable influence over the whole of the respiratory structure, productive of sympathetic effects, so irritative of the laryngeal affection, as sometimes to be mistaken for it.

Certain forms of bronchitis have also a resemblance to laryngitis—though independently of other differances, they are distinguishable by the heavy thoracic oppression, the wheezing and rattling, the œdematous face, and the clear and colorless lips, in the intervals of the paroxysms. But here, as well as in phthisis pulmonalis, and most other diseases of the lungs, all doubt and obscurity may be removed by external exploration.

Nor should it be forgotten, in these investigations, that a set of phenomena, very closely simulating real laryngitis, may be owing to nervous and muscular states, influencing the vocal mechanism.

Cases of this kind, though easily detected, I have seen, from carelessness, so frequently confounded, that I deem it proper to direct attention to them.

Great pains have been taken in reference to the discrimination between the laryngeal and tracheal affections, and from the general similarity of their aspect, it is not always readily accomplished. Except as to an operation, hereafter to be noticed, it is fortunate in a practical relation, that it is not very material. But some precision is attainable in this respect, by attention to the seat of the painful sensations, to the greater alteration of the voice, in the laryngeal lesion, to the nature of the cough, which is preceded by tickling, and is more stridulous and spasmodic, and very constantly brought on, by any thing irritating applied to the fauces, as gargle, or even drinks, or food of this description.

In our prognostications of the event, we must be unavoidably perplexed by the stage of the case, the various conditions of the larynx, which cannot always, with any exactness be appreciated, by any method of determination and equally so by the existing diathesis. Taking the mildest specimen of the disease, when fully established, and in other particulars of the most favorable character, the cure is exceedingly difficult and uncertain. Even a mere thickening of tissues from ordinary inflammation is seldom removed, and the case being dependent on a strumous, and still more on a tubercular condition, it is nearly hopeless. On the whole, I have experienced much less difficulty in relieving those attacks which have come on as sore throats, and where this has continued to form the most prominent feature of the case, the affection of the larynx probably consisting in little else than a mere sympathetic irritation.

An autopsic inspection sometimes shews little or no inflammation in the mucous membrane, but usually otherwise, the most usual change being that of thickening, having on its surface granulations, or small ulcers, particularly around the glottis, or within its cavity. Lesions of this sort may indeed, be very extensive, even to the destruction of the epiglottis, and its immediate connections. Conversions too, of the cartilages are met with, into gritty calcareous matter, mixed up with portions of denuded and carious bone, or mortified, black, and dissolved, so as to resemble wet rotten leather. Cutting into the sub-cellular membrane, it is sometimes dense and solid, its reticulated texture obliterated, and as it were incorporated with the mucous tissue. Œdema of it, when the structure is preserved, is not uncommon, or in place of serous effusion, pus is infiltrated through it, or collected in small sacculi, or abscesses.

* In the lecture on the affections of the throat.

The trachea may be also granulated, or ulcerated, while in other instances, the whole respiratory tube, the upper and lower section, contains tubercles separate, or combined with the same productions in the lungs. Besides the latter, however, which may be incipient formations, or in the several stages to absolute maturity, the lungs have sometimes been observed in various other modes affected, and especially by effusions into their cellular texture.

Of the phenomena in the throat, I have only to say, that there may be no appreciable change on those already described, or ulceration more or less extensive and modified, as originating from mercury, syphilis, scrotula, or other causes.

My pathological observations on this subject will be concise. It appears that the affections arranged under the generic title of chronic laryngitis, as indeed I have already intimated, are diversified, seated in the mucous, or sub cellular tissue, of one or the other portion, or the whole of the respiratory tube, sometimes separately, on other occasions in both ligaments, arising from inflammation originating in these, or derived by an extension of it from adjacent strictures, induced by various causes, operating directly or indirectly, ending in the organic lesions of the parts immediately concerned, or with the several complications previously mentioned.

*(To be concluded in our next.)*

